# What’s for lunch? The content and quality of lunches consumed by Dutch primary schoolchildren and the differences between lunches consumed at home and at school

**DOI:** 10.1186/s12889-019-7750-9

**Published:** 2019-10-24

**Authors:** Frédérique C. Rongen, Ellen van Kleef, Sienna Sanjaya, Monique H. Vingerhoeds, Elly J. M. Buurma-Rethans, Coline van den Bogaard, Caroline T. M. van Rossum, Jacob C. Seidell, S. Coosje Dijkstra

**Affiliations:** 1 Department of Health Sciences, Faculty of Science, Vrije Universiteit Amsterdam, Amsterdam Public Health research institute, De Boelelaan 1085, 1081 HV Amsterdam, The Netherlands; 2 Marketing and Consumer Behaviour Group, Wageningen University, Wageningen, The Netherlands; 3 Food, Health & Consumer Research group, Wageningen Food & Biobased Research, P.O. Box 17, 6700AA Wageningen, The Netherlands; 4 Centre for Nutrition, Prevention and Health Services, RIVM, Bilthoven, The Netherlands

**Keywords:** Lunch, Children, Primary school, Diet

## Abstract

**Background:**

Lunch is an important part of a healthy diet, which is essential for the development, growth and academic performance of school-aged children. Currently there is an increasing number of Dutch primary schoolchildren who are transitioning from eating lunch at home to school. There is limited knowledge about the current quality of the lunches consumed by primary schoolchildren in the Netherlands and whether there are any differences between lunches consumed at home or at school. To investigate differences in content and quality of lunches consumed by Dutch primary schoolchildren at home and at school.

**Methods:**

Cross-sectional study among 363 Dutch primary schoolchildren aged 4–12 years based on the first two years of the 2012–2016 Dutch National Food Consumption Survey. Demographic characteristics were obtained through a questionnaire. Diet was assessed with two non-consecutive 24-h dietary recalls. Quality of lunches was assessed on their nutritional quality whether they fitted the nutritional guidelines. ‘Nonparametric tests were used to examine the content and quality of the lunches between place of consumption and parental educational position.

**Results:**

The most consumed lunch products among primary schoolchildren were bread, dairy products and sugar-sweetened beverages. Fruit and vegetable consumption was very low. Consumption of milk and other dairy products was higher among children who eat lunch at home than children who eat lunch at school (*p* < 0.01). Consumption of sugar-sweetened beverages was higher among children who eat lunch at school than children who eat lunch at home (p < 0.01), and at school a higher proportion of the drinks did not fit within the Dutch dietary recommendations (p < 0.01).

**Conclusions:**

The current content of the lunches consumed by Dutch primary schoolchildren leaves room for improvement, especially regarding fruit and vegetables. The statistically significantly higher consumption of sugar-sweetened beverages and lower consumption of milk and dairy products at school vs. home is worrisome, as currently more children in the Netherlands are transitioning to having lunch at school.

## Background

Childhood obesity and overweight have reached epidemic levels in many countries around the world [1]. In 2014, the World Health Organization (WHO) reported that one in every three European children between the ages of 6 and 9 is either overweight or obese [2]. In the Netherlands, 16.1% of children aged 4 to 11 are considered to be overweight or obese [3]. The increasing proportion of overweight and obese Dutch children over recent decades has made this a major public health concern [4].

One of the behavioral factors associated with childhood overweight and obesity is the frequent consumption of foods and beverages that are high in calories and low in nutritional value [5]. Developing healthy eating behaviors during childhood is important because they are likely to be maintained through adulthood [6]. Unhealthy eating patterns and habits formed during childhood are related to non-communicable diseases (such as diabetes, osteoporosis and hypertension) [7]. There is also evidence that healthy eating habits are positively associated with improved academic performance of children [8–10]. These advantages stress the importance of establishing healthy eating behaviors during childhood.

Given that school-aged children spend most of their time at school, numerous childhood overweight and obesity prevention programs have focused on school-based interventions, such as teachings strategies [11], school gardens [12] and school lunch programs [13, 14]. Unlike in many other countries, primary schools in the Netherlands do not provide lunches. As a result, the content of the lunches consumed by children at school is primarily the responsibility of parents [15]. Dutch children typically have a 60–90 min lunch break, during which they go home to eat their lunch or eat home-packed lunches at school. It is not common that Dutch children consume a hot or cooked meal during lunch at home or at school. Primary school children in the Netherlands do not have the opportunity to purchase foods or drinks at school. There are no vending machines, school cafeteria’s or school canteens present. Lunch mostly contains sandwiches with water, milk or a sugar-sweetened beverage, and sometimes candy or biscuits [15, 16]. Fruit and vegetables are typically not part of the lunch [16, 17]. This corresponds with findings in other countries, where although content varies, overall lunch boxes also lack fruit and vegetables and often contain sugar-sweetened beverages and snacks [18, 19]. In the Netherlands, there has been an increasing number of schools during the past few years who have switched to what is called a “continuous schedule” that shortens the school day and allows for a 30-min lunch and outdoor playing break at school [20]. All children are expected to bring a home-packed lunch and a drink, to be consumed during this half-hour lunch break. Therefore, it is relevant to investigate if there are differences between lunches consumed at home or at school. This information can be used for the development of interventions or policies to stimulate healthy eating at school.

It has been shown that unhealthy dietary behavior is more prevalent among groups with a low socio-economic position (SEP) [21, 22]. In general, people with a lower SEP are more likely to have a higher intake of energy, sugar, salt and saturated fat and a lower intake of fruit, vegetables and fish than people with a higher SEP [21, 23–25]. The relatively high price of healthier food may keep children from families with a relatively low SEP from healthy eating behavior [26, 27].

Considering the current transition in the Netherlands from eating lunch at home to eating lunch at school, it is of interest to understand whether there are differences in the content and quality of lunches consumed at both places. To our knowledge, no previous study has reported on the differences between the current lunch of primary schoolchildren at home and at school. Hence the objective of the present study is to investigate the content and quality of lunches consumed by Dutch primary schoolchildren aged 4 to 12 on schooldays. We also examined the role of SEP in the difference between lunch consumption at home and at school.

## Methods

### Study design, procedure and sample

Data from the first two years of the Dutch National Food Consumption Survey (DNFCS) 2012–2016 were used, which estimated dietary intake from two non-consecutive 24-h recalls among Dutch children and adults aged 1–79 years. Details of this representative cross-sectional study are described elsewhere [16]. In brief: subjects were drawn from representative consumer panels of a large Dutch data and research company [28]. Information such as current highest educational level of both parents, native country of parents and child, and general dietary information of the child were collected through a questionnaire sent by mail or distributed online via the marketing agency’s website prior to an interview/24-h recall after written consent was obtained from the parents of the participants. Two different age-specific questionnaires were used for children aged 4–11 years and 12–18 years. For children ages 4–11 years parents filled out the questionnaire for their children. Children aged 12–18 years filled out the questionnaire by themselves.

**Fig. 1:**
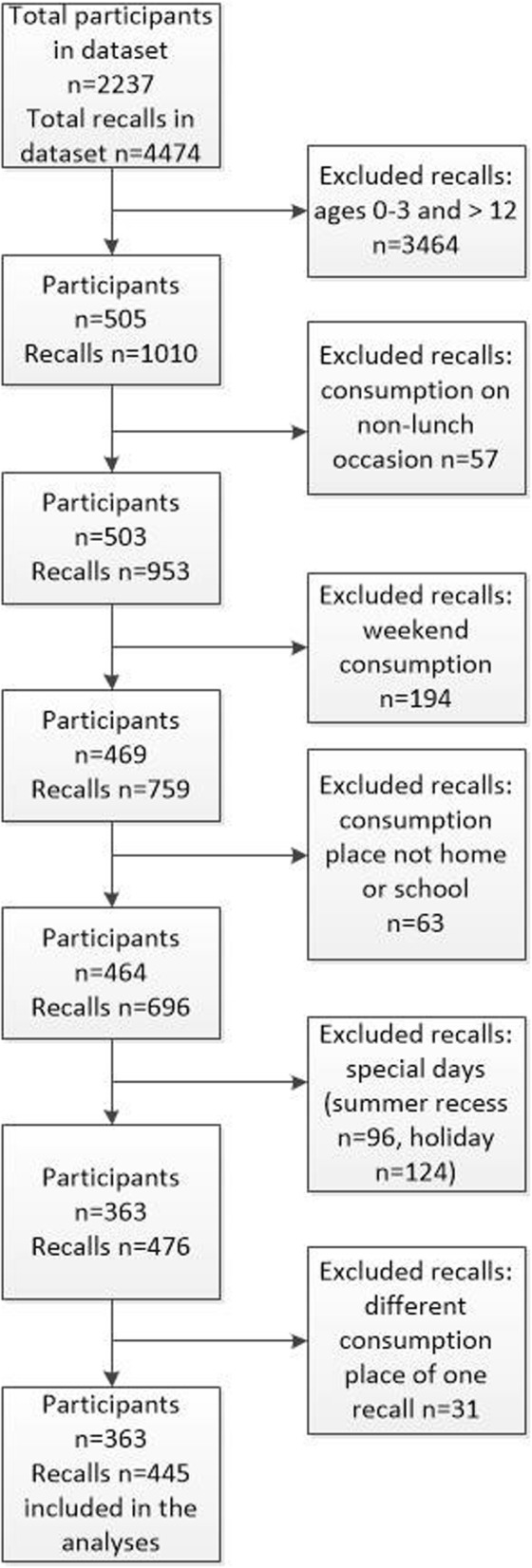
Flowchart excluding recalls

The present study was based on data of the 4–12 age group, comprising a sample of 505 participants (Fig. 1). As this study focuses on the lunches consumed at school and at home, recalls without dietary intake at the lunch food occasion (*n* = 57) and lunches consumed at other locations (e.g. restaurants) (*n* = 63) were excluded. Data collections conducted for consumption on weekends (*n* = 194) and summer recess were also excluded (*n* = 96). Another 124 recalls on a special-event day (Easter, Christmas) were excluded. Lastly, 31 children had two recalls because the location of the lunch consumption was different – one recall was at home and the other recall at school. For these children, only the data and place of consumption from the first recall was used and the second recall was excluded (*n* = 31). Under the mentioned conditions, 363 Dutch primary schoolchildren were included in the final analyses.

### Dietary assessment and intake

Dietary intake data were collected with two 24-h dietary recalls. Parents/caretakers of children aged 1 to 8 years were invited to complete a food diary of their child on two specific days, also covering the consumption at daycare, school or elsewhere. Interviewers gave instructions by telephone. The day after the first registration of consumption in the food diary, a face-to-face interview with the parent/caretaker was conducted during a home visit; children 4–8 years were present during the interview. During this visit both height and weight were measured. The second 24-h recall was conducted by telephone. Both dietary recalls were conducted by trained dieticians on independent days about six weeks apart. For children aged 9–15, the two 24-h recalls were conducted by means of two face-to-face interviews during home visits. Contact with children aged 9–15 was made initially through a parent or caretaker; the interviews were conducted with the child in the presence of the parent or caretaker. During the first visit both height and weight of the children were measured*.*

At the interview, time of consumption for each consumption moment during the recalls was recorded and divided into four categories: breakfast, lunch, dinner and in-between meals. The location of consumption was established for each food consumption moment. The season in which the first recall was conducted was recorded during the interview and categorized into summer, winter, spring and autumn. In addition, general dietary information covered usual eating habits, dietary restrictions and frequency of consumption of specific foods such as fish and vegetables. The computer-directed interview program EPIC-Soft (©IARC) was used to guide and record the dietary recalls ensuring standardized data collection [29]. Detailed description and quantification of the foods consumed were recorded. Standardized means were used to quantify the portion sizes, such as standard units, weight/ volume, pictures, or household measures [5].

Dietary intake of 82 children was calculated by average consumption over the two 24-h recalls. For 281 children (77.4%), there was only one eligible recall due to elimination based on the exclusion criteria.

### Content and quality of lunchboxes

Dietary intake of the children was classified into food groups. The food group classification was developed in collaboration with the Netherlands Nutrition Center and based on the Food choices guidelines of the Wheel of Five [30]. The Wheel of Five is the practical information tool used by the Netherlands Nutrition Center to provide examples of healthier options in five food groups, based on nutrient content for product [31]. For instance, for the food group “drinks” water is categorized within the Wheel of Five and lemonade is categorized outside it. Based on food group we classified the lunches into 10 main food groups, including bread and cereal products, butter and margarine, processed meats, cheese/cheese products, other bread toppings and spreads, milk and other dairy products, drinks (e.g. water, lemonade), vegetables, fruit/fruit products, and snacks. Percentage of users and amounts converted into grams (g) of all food groups were calculated. Food groups that had a user percentage < 5 were not taken into account (e.g. fish, egg/egg products, nuts, soups, condiments). Quality of the lunch contents was assessed by calculating which percentage of consumed products in the main food groups consists of recommended products in the Wheel of Five.

### Place of consumption of the lunch

During the interview the location of consumption was established for each food consumption moment. There were 10 possible locations respondents could choose from: home, workplace, school, friends/family/caretaker, sports center, outside (street, park and market), traveling (bicycle, car), restaurant, shop/hairdresser/church, and other. For this study only the locations home, school and family/friends/caretaker were included, as these are regular lunch places on schooldays. Place of consumption was divided into two groups “at home” or “at school”. “At home” was based on the locations “home” and “family/friends/caretaker” as the eating habits and products consumed at lunch will probably be the same. “At school” was based on the location “school”.

### Educational levels of the parents

Parents’ educational level was assessed with the question: what is the highest level of education you and your partner have completed? Respondents could choose from seven possible answers: primary education, lower vocational education, advanced elementary education, intermediate vocational education, higher general vocational education, higher vocational education, and university. The highest level of one of the parents was used to classify them into the three educational levels. A high education level referred to university and graduation from higher vocational school. Middle education level referred to a background with higher general or intermediate vocational education. Low education level referred to the highest completed level of education such as primary school, lower or intermediate general education, and lower vocational school.

### Characteristics

Degree of urbanization was distinguished into low (< 1000 addresses/ km^2^), moderate (1000–1500 addresses/ km^2^), and high (> 1500 addresses/ km^2^). The migration background was assessed with the question, “What is the country of birth of the participating child and his/her biological parents?” with seven possible answers: the Netherlands, Surinam, Netherlands Antilles, Aruba, Turkey, Morocco, and other. This category was later split into “Western” and “Non-western”. A Western background includes Europe (excluding Turkey), North America, Oceania, Indonesia or Japan, while any other country is a Non-western background [32]. To classify the background of the child the migration background of the mother is leading, except when the mother had a Dutch background, in which case the migration background of the father was used. When the background was known for only one of the parents then the background of this parent was used. When the migration background of both parents was unknown, the background of the child was marked as unknown. Measured height and weight were used to calculate body mass index (BMI) in kg/m^2^. Age- and gender-specific cut-off values were utilized for BMI classification [33].

### Statistical analyses

Descriptive analyses were used to summarize participants’ demographic characteristics and the content of lunches consumed (total and separated by place of consumption). Because the distribution of the data was skewed, the median of daily consumption in grams was presented along with the 25th and 75th percentiles. Differences in percentage of users of the food groups for lunches consumed at home and at school were calculated with a Chi-Square test. Differences in the number of lunches per food group in grams between lunches consumed at home and at school were calculated with a Mann-Whitney U-test.

Differences in educational level were tested with a Kruskal-Wallis analysis. This test was conducted to assess the differences in lunch contents in grams for children who consume their lunch either at home or at school among the three educational levels (low, middle and high). When the results were statistically significant, post-hoc analysis were done to identify which pair(s) of groups differed. A probability value ≤0.05 was considered to be statistically significant. All analyses were done using Statistical Package for the Social Sciences SPSS v 23.0 for Windows [34].

## Results

### Participant characteristics

The characteristics of the participants are presented in Table 1. A total of 363 children were included in the present study, distributed equally by gender (49.9% girls). Average age was 7.9 years (*SD* = 2.6). Almost all of the participants had a Western background (93.1%). The vast majority of the children had a normal BMI (82.4%). Almost half of the parents had a high level of education (48.5%), followed by 41% with mid-level education and 10.5% with low-level education. Of the included children, place of consumption of the 24-h recall was 51% at home and 49% at school. Boys (56.8%) consumed their lunch statistically significantly more often at home than girls (43.3%). No other statistically significant differences were observed for characteristics or place of consumption.

**Table 1:** Characteristics of Dutch primary schoolchildren aged 4–12 in the Dutch National Food Consumption Survey 2012–2014 in total and separated by place of consumption (at home or at school)

	Total (*n* = 363)		Place of consumption			
			Home (*n* = 185)		School (*n* = 178)	
	n	%	n	%	n	%
Gender						
Boys	182	50.1	105	56.8*	77	43.3*
Girls	181	49.9	80	43.2*	101	56.7*
Age (years)						
4–6	121	33.3	67	36.2	54	30.3
7–9	128	35.3	65	35.1	63	35.4
10–12	114	31.4	53	28.6	61	34.3
Urbanization						
Low	156	43.0	76	41.1	80	44.9
Moderate	76	20.9	42	22.7	34	19.1
High	131	36.1	67	36.2	64	36.0
Migration background						
Western	338	93.1	174	94.1	164	92.1
Non-western	25	6.9	11	5.9	14	7.9
BMI categories						
Normal	299	82.4	152	82.2	147	82.6
Overweight/ obese	64	17.7	33	17.8	31	17.4
Parents’ highest educational level						
Low	38	10.5	17	9.2	21	11.8
Middle	149	41.0	84	45.4	65	36.5
High	176	48.5	84	45.4	92	51.7
Season of measurement						
Spring	96	26.4	54	29.2	42	23.6
Summer	65	17.9	31	16.8	34	19.1
Autumn	102	28.1	49	26.5	53	29.8
Winter	100	27.5	51	27.6	49	27.5
Number of recalls						
1	281	77.4	152	82.2	129	72.5
2	82	22.6	33	17.8	49	27.5
Day of consumption						
Monday	62	17.1	26	14.1	36	20.2
Tuesday	92	25.3	38	20.5	54	30.3
Wednesday	64	17.6	52	28.1	12	6.7
Thursday	84	23.1	40	21.6	44	24.7
Friday	61	16.8	29	15.7	32	18.0

*BMI* Body mass index

*Statistically significant differences in percentage of boys and girls between the groups at home and at school (*p* ≤ 0.05)

### Content of the lunches

Table 2 shows the content of lunches per food group in total and divided by place of consumption. Almost all children (98.4%) consumed bread or cereal products for lunch. The median intake was 70 g, the equivalent of two slices of bread. Most children used butter or margarine on their lunchtime bread (71.1%). Processed meats and sweet spreads were most frequently consumed as bread toppings (51.5 and 49.3% respectively). Cheese as a bread topping was consumed by 31.4% of the children. Most-frequently consumed beverages were milk and other dairy drinks (44.4%) and non-dairy drinks (58.7%) (e.g. water 15.7%, fruit juice 5.5% and sugar-sweetened beverages 40.8%). Vegetables (6.9%) and fruit/fruit products (8.0%) were only consumed by a small proportion of the children at lunch. Almost a quarter (23.1%) of the children consumed a snack.

**Table 2:** Content of lunches per food group of Dutch primary schoolchildren in the Dutch National Food Consumption Survey 2012–2014, total and separated by place of consumption (at home or at school)

	Total (n = 363)					Place of consumption									
						Home (*n* = 185)					School (*n* = 178)				
	Users		Median	P25	P75	Users		Median	P25	P75	Users		Median	P25	P75
	n	%	g	g	g	n	%	g	g	g	n	%	g	g	g
Bread and cereal products	357	98.3	70.0	49.4	82.5	181	97.8	70.0	45.0	88.8	176	98.9	70.0	52.5	70.0
Butter and margarine	258	71.1	6.0	0.0	12.0	131	70.8	6.0	0.0	12.0	127	71.4	6.0	0.0	9.6
Processed meats	187	51.5	5.0	0.0	16.5	97	52.4	6.5	0.0	16.0	90	50.6	4.0	0.0	16.7
Cheese/cheese products	114	31.4	0.0	0.0	11.1	60	32.4	0.0	0.0	12.0	54	30.3	0.0	0.0	7.5
Other bread toppings and spreads (e.g. jam, honey, chocolate sprinkles)	179	49.3	0.0	0.0	15.4	91	49.2	0.0	0.0	18.0	88	49.4	0.0	0.0	14.1
Milk and other dairy products	161	44.4	0.0	0.0	190.8	109	58.9*	137.3	0.0	202.9	52	29.2*	0.0	0.0	150.4
Drinks	213	58.7	149.0	0.0	210.0	83	44.9*	0.0	0.0	199.8	130	73.0*	200.0	0.0	246.5
Water/tea	57	15.7	0.0	0.0	0.0	33	17.8	0.0	0.0	0.0	24	13.5	0.0	0.0	0.0
Fruit juice	20	5.5	0.0	0.0	0.0	8	4.3	0.0	0.0	0.0	12	6.7	0.0	0.0	0.0
Sugar-sweetened beverages	148	40.8	0.0	0.0	200.0	49	26.5*	0.0	0.0	81.1	99	55.6*	103.1	0.0	214.5
Vegetables	25	6.9	0.0	0.0	0.0	13	7.0	0.0	0.0	0.0	12	6.7	0.0	0.0	0.0
Fruit/fruit products	29	8.0	0.0	0.0	0.0	12	6.5	0.0	0.0	0.0	17	9.6	0.0	0.0	0.0
Snacks	84	23.1	0.0	0.0	0.0	42	22.7	0.0	0.0	0.0	42	23.6	0.0	0.0	0.0

P25, 25th percentile, p75, 75th percentile

*Statistically significant differences in percentage of users between the groups at home and at school

Children who ate lunch at home consumed milk and other dairy products statistically significantly more often (58.9%) than children who ate lunch at school (29.2%), *p* < 0.01. Children who ate lunch at school consumed sugar-sweetened beverages statistically significantly more often (55.6%) than children who ate lunch at home (26.5%), *p* < 0.01.

Results of the nonparametric analyses are presented in Table 3. Consumption of other toppings and spreads was statistically significantly higher in children who ate lunch at home (*median* = 18.1 g) compared to children who ate lunch at school (*median* = 14.2 g), *U* = 3245.5, *p =* 0.029. Children who ate lunch at school had a statistically significantly higher consumption of drinks (*median* = 206.0 g) than children who ate lunch at home (*median* = 200.0 g), *U* = 4534.0, *p* = 0.049.

**Table 3:** Comparison amount (g) consumed of food groups during lunch of primary schoolchildren based on place of consumption as reported in the Dutch National Food Consumption Survey (2012–2014)

	Place of consumption				
Food groups	Median (g)		*U*	*z*	*p*
	At home (n = 185)	At school (n = 178)			
Bread and cereal products	70.0	70.0	14,954.5	−1.02	0.309
Butter and margarine	9.0	8.0	7595.5	−1.22	0.224
Meat products	16.0	16.5	4061.0	−.83	0.409
Cheese	18.0	15.4	1553.5	−.38	0.705
Other toppings and spreads	18.1	14.2	3245.5	−2.19	0.029*
Milk and other dairy products	190.8	206.0	2337.0	−1.80	0.072
Drinks	200.0	206.0	4534.0	−1.97	0.049*
Vegetables	39.7	25.8	57.5	−1.12	0.265
Fruit/fruit products	82.2	67.3	71.5	−1.35	0.177
Snacks	19.0	12.5	666.5	−1.93	0.054

Mann-Whitney U-analysis. *p ≤ 0.05

### Percentage of products recommended in the wheel of five

**Fig. 2:**
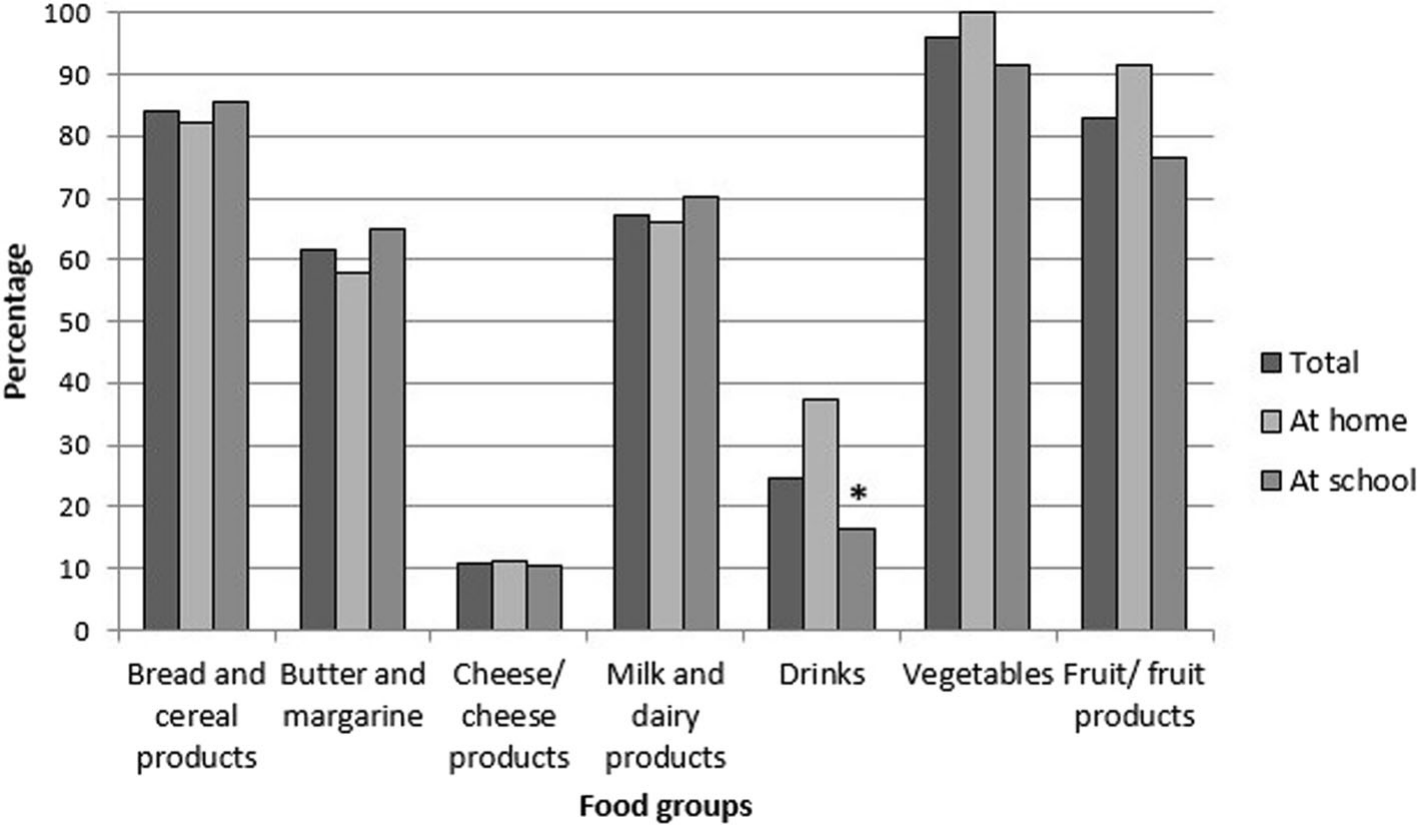
Percentages of consumed foods recommended in the Wheel of Five per food group** and location. Legends: * Statistically significant differences in percentages within the Wheel of Five at home and at school. **Product groups not containing any Wheel of Five products are not presented (processed meats, other bread toppings, spreads and snacks)

Figure 2 shows the percentage of products that are recommended in the Wheel of Five of the Netherlands Nutrition Center per food group of the total amount consumed by the children. All foods consumed were assessed on their nutritional quality by determining whether they fitted the nutritional guidelines of the Wheel of Five. For instance, 8% of the children consumed fruit or fruit products and of which more than 80% were recommended in the Wheel of Five. Children who ate lunch at home had a statistically significant higher proportion of drinks (37.3%) recommend in the Wheel of Five (*p* < 0.01) than children who ate lunch at school (16.4%). No statistically significant differences were found for the other food groups (bread and cereal products, butter and margarine, cheese/cheese products, milk and dairy products, vegetables, and fruit/fruit products).

### Content of lunches by educational levels of parents

A Kruskal-Wallis test was conducted to investigate differences among the three educational levels (low, middle and high) and the content of the lunches in grams for children who ate lunch at home and at school. For the children who ate lunch at school there was a statistically significant difference in “milk and other dairy products” between educational levels, X^2^ (2) = 6.43, *p* = 0.04. Other product groups showed no statistically significant differences between parental educational levels for children who ate lunch at school.

A post-hoc analysis was conducted within the three educational levels to evaluate the pairwise differences. The result of these tests indicate that children of at least one parent with a high educational level consumed statistically more of the “milk and other dairy products” group than their counterparts in the lower educational level who ate lunch at school X^2^ (2) = 6.43, *p* = 0.04. For children who ate lunch at home there were no statistically significant differences between parental educational level and lunch contents.

## Discussion

The present cross-sectional study of Dutch primary schoolchildren assessed the content and quality of lunches consumed on schooldays at home and at school. The descriptive analysis showed that the typical lunch for Dutch primary schoolchildren is a sandwich with a sweet or savory topping and a drink (milk or sugar-sweetened beverages). Intake of fruit and vegetables at lunch is very low, while there is a high consumption of sugar-sweetened beverages. This study showed that milk and other dairy products were statistically significantly more likely to be consumed at lunch at home while consumption of sugar-sweetened beverages was significantly higher among children eating lunch at school. Hence lunchtime drinks consumed at school were less healthy (e.g. fruit juices and sugar-sweetened beverages) than drinks consumed at home.

The results showed that most Dutch primary schoolchildren do not eat fruit and vegetables for lunch. In fact, only 7% of children consume vegetables for lunch. The Wheel of Five recommends at least 150 g of fruit and 100–150 g of vegetables per day for children aged 4–8, and at least 200 g of fruit and 150–200 g of vegetables per day for children age 9–13 in order to reduce the risk of cardiovascular disease [31]. Our results are comparable with those of Rees et al., in that only 8% of the packed lunches contained at least one portion of vegetables/salad/beans [35]. The findings are also in line with the most recent food consumption survey in the Netherlands that showed that approximately 60% of the children do not consume the recommended amount of fruit (200 g) and vegetables (150–200 g) per day [36]. An explanation for the low intake of fruit and vegetables at lunch is the absence of these food groups from Dutch lunch culture [37]. In the Netherlands vegetables are typically consumed in the evening warm meal [16, 38], while fruits are usually eaten during the morning break or after school [39]. Eating fruit and vegetables for lunch will therefore require a major change in behavior. Previous research on behavioral changes in consumption of fruit and vegetables among school-aged children emphasizes that the environment has a strong influence on food consumption. In particular, making fruit and vegetables available at moments other than the evening meal will increase consumption [39]. A review of Hendrie et al. states that repeated exposure to vegetables is linked to short-term behavioral change [40]. The school setting can thus provide an opportunity to expose children more to fruit and vegetables.

The data suggest that children who eat lunch at school replace dairy products with other drinks, such as sugar-sweetened beverages. Concerning the differences in dietary intake and nutritional quality of lunches consumed at home or at school, the food group “milk and other dairy products” was more likely to be consumed at home, while the food group “all other drinks” was more likely to be consumed at school. Lunchtime consumption of milk and other dairy products is 60% at home and 30% at school. Not only the quantity is important, but also the quality of the milk and dairy products. Many dairy products have a high sugar content and are not recommended in the Wheel of Five. The data did show, however, that those children who consume milk and dairy products at school consume 70% of the products recommended in the Wheel of Five – which is slightly more than children who eat lunch at home, where 66% of the products are recommended in the Wheel of Five. The difference in consumption may be explained by the fact that dairy, as a perishable product requiring refrigeration, is not seen as a suitable drink to pack in the morning for a school lunch. Parents usually take the children’s preferences into consideration when packing their lunch, which explains the high prevalence of juice and soft-drink consumption [39]. This result is worrisome, as there is an increasing number of primary schools who are transitioning to a continuous schedule [20]. Milk was the most consumed product in the “milk and other dairy products” group of home-consumed lunches, and its sugar content is often lower than that of juices and carbonated drinks. Various studies have highlighted the association between high consumption of sugar-sweetened beverages and increased risk of overweight and obesity in children [41–43].

Parental educational level did not influence the content and quality of lunches, except that children of higher-educated parents who eat lunch at school consume more milk and other dairy products than children of less-educated parents. Although in previous studies parental educational level has been linked to children’s consumption of food groups, such as sugar-sweetened beverages (i.e. fruit juices and carbonated drinks), meat, poultry and fats [44–46], this relation was not observed in the current data. This might be the result of the present study focusing solely on lunch consumption during schooldays. It may yield less variation in dietary intakes (almost all children were eating sandwiches for lunch) and consumption of many food groups was very low, therefore the data did not provide sufficient statistical power to detect differences.

This is the first study to focus uniquely on the quality of lunch among primary schoolchildren, assessing the differences between lunches consumed at school and at home during schooldays in the Netherlands. Strengths of the DNFCS 2012–2016 was that it is conducted among a representative sample of the Dutch population and the use of 24-h recalls. However, only the first two years of the data were analyzed because the last two years were not available yet. Several limitations of this study must be taken into account. First, some methodological considerations need to be acknowledged. Nonparametric analyses were used to analyze the differences between places of consumption and parental educational levels. A disadvantage of this method is that it is not possible to adjust for potential confounders, such as some demographic characteristics and season; the results of this analysis should therefore be interpreted with caution. Second, the cross-sectional nature of the study does not allow causal association to be established between the variables. Third, information collected in the present study is based on self-reported data, including background information and dietary recalls based on a food diary. This method may affect the validity of the information given, as many factors could influence the response given by children or parents. Fourth, due to the limitation of number of study participants we did not analyzed subgroups for age and would be of interest to include in future research. In this study time constraints of working parents is not taken into account and could be associated with less adequate nutrition habits of children. For future research it would be interesting to include this to provide more insight for policy makers.

The findings from the current study nonetheless provide some insights on improvements regarding fruit and vegetable intake and consumption of sugar-sweetened beverages that can be implemented to increase the quality of lunches consumed by Dutch primary schoolchildren. School environment has been recognized as one factor that can influence children’s eating habits [15, 37]. A school food policy on lunches is a practical component of promoting healthy eating habits among children [47]. Since most Dutch primary schools do not provide school lunches, policies are needed in order to regulate issues concerning foods and beverages brought to school. School food policies should focus on increasing the consumption of fruit and vegetables, stimulating consumption of water and milk, and decreasing the consumption of sugar-sweetened beverages. The review of Micha et al. showed that school food environment policies have a positive effect on fruit and vegetable intake and reduce the intake of sugar-sweetened beverages [48]. The Dutch government can also play a role in encouraging and supporting schools to implement a national school lunch program. An intervention study by Andersen et al. (2014) among 834 Danish children found significantly higher consumption of fruit and vegetables after replacing their usual packed lunch with school meals [49]. Other studies show that school lunches improve the total dietary quality and nutritional intake of children [50, 51], and lead to higher consumption of fruit and vegetables [52].

## Conclusion

The results of the present study demonstrate that the current dietary intake and the quality of lunches consumed by Dutch primary schoolchildren leave substantial room for improvement, especially when it comes to the low intake of fruit and vegetables and the high intake of sugar-sweetened beverages. The higher consumption of sugar-sweetened beverages among children who eat at school in comparison with children who eat at home is worrisome, as more children are transitioning to eating lunch at school. Schools have a great potential in contributing to resolve these issues by increasing the awareness, availability and accessibility of healthy foods, as well as promoting healthy eating behaviors among children and parents. Current findings also highlight the importance of future research investigating the appropriate component to increase fruit and vegetable intake while decreasing the consumption of sugar-sweetened beverages when eating lunch at school. It is also crucial to investigate whether school food policy or implementation of a school lunch program could improve the aforementioned conditions.

## Data Availability

The data that support the findings of this study are available from Centre for Nutrition, Prevention and Health Services - RIVM but restrictions apply to the availability of these data, which were used under license for the current study. Data can be requested directly the from the Centre for Nutrition, Prevention and Health Services – RIVM, www.wateetnederland.nl.

## Abbreviations

DNFCS: Dutch National Food Consumption Survey
SEP: Social Economic Position
WHO: World Health Organization
